# Laparoscopic inguinal ligament suspension versus laparoscopic sacrocolpopexy in the treatment of pelvic organ prolapse: study protocol for a randomized controlled trial

**DOI:** 10.1186/s13063-018-2494-x

**Published:** 2018-03-05

**Authors:** Chunbo Li, Zhiyuan Dai, Huimin Shu

**Affiliations:** Department of Gynaecology and Obstetrics, Shanghai First Maternity and Infant Hospital, Tongji University School of Medicine, 536 Changle Road, Shanghai, China

**Keywords:** Pelvic organ prolapse, Laparoscopic inguinal ligament suspension, Laparoscopic sacrohysteropexy

## Abstract

**Background:**

Pelvic organ prolapse (POP) is a common health problem. The lifetime risk of undergoing surgery for prolapse is 11%. POP significantly affects the effects on quality of life and activities of daily living. Laparoscopic sacrocolpopexy (LSC) has been viewed as the gold standard treatment for women with POP who desire reconstructive surgery. However, LSC is associated with technical difficulties, resulting in a long learning curve and operative time. Recently, our team introduced a new laparoscopic technique of inguinal ligament suspension (LILS) and had confirmed its safety and efficacy in treating vaginal vault prolapse. As a new surgical technique for POP, a prospective randomized controlled trial comparing the LILS with the standard technique of LSC is necessary. Therefore, we will conduct a trial.

**Methods:**

The trial is a randomized controlled trial. It compares LILS with LSC in women with stage 2 or higher uterine prolapse. The primary outcomes of this study are perioperative parameters, including surgical time, blood loss, intraoperative complications, and hospital stay as well as surgical anatomical results using the pelvic organ prolapse questionnaire (POP-Q) classification at 6 weeks, 6 months, 12 months, and annually till 5 years after surgery. Secondary outcomes are subjective improvement in urogenital symptoms and quality of life, postoperative complications, postoperative recovery, sexual functioning, and cost-effectiveness at each follow-up point. Validated questionnaires will be used and the data will be analyzed according to the intention-to-treat principle. Based on an objective success rate of 90%, a noninferiority margin of 15%, and a dropout of 20%, 107 patients are needed in each arm to prove the hypothesis with a 95% confidence interval.

**Discussion:**

The trial is a randomized controlled, multicenter, noninferiority trial that will provide evidence whether the efficacy and safety of LILS is noninferior to LSC in women with symptomatic stage 2 or higher uterine prolapse.

**Trial registration:**

China Trial Register (CTR): ChiCTR-INR-15007408. Registered on 9 November 2015.

**Electronic supplementary material:**

The online version of this article (10.1186/s13063-018-2494-x) contains supplementary material, which is available to authorized users.

## Background

Pelvic organ prolapse (POP) is a common condition that negatively affects substantial discomfort, quality of life and activities of daily living of up to 40% of all women [1]. With the aging, informed, and active female population, there is a growing need for high-quality and cost-effective treatment options for POP [2]. While various procedures have been designed to treat POP, until now the most successful method has been laparoscopic sacrocolpopexy (LSC), with overall objective anatomical and subjective success rates of 92% and 94.4%, respectively [3]. In this procedure, a mesh graft is used to attach the anterior and posterior walls of the vagina to the anterior longitudinal ligament at the sacral promontory. It provides an excellent cure rate, durability and low morbidity, and has been considered to be the gold standard procedure for POP [3–5].

However, LSC is technically more challenging because it requires surgical skills to perform suturing with knot tying. In addition, dissection to safely access the anterior longitudinal ligament at the level of the promontory may be a challenge, particularly for obese women, dense abdominopelvic cavity adhesions, large common iliac arteries, or when an anatomical variation exists. The procedure usually involves, in various deficiencies such as intrusion to the area of the retroperitoneal nerve plexuses, nonphysiologic placement of the vagina, and attachment to the sacrum associated with certain risks of vascular complications [6]. Thus, the technique is usually reserved for experienced surgeons who have excellent orientation in the anatomical structures of the lesser pelvis with particular focus on the rectovaginal and retroperitoneal pelvic floor space. These factors limit the popularization of this technique.

The inguinal ligament is a very important structure for surgery; it acts as a strong anchor or origin for the abdominal wall musculoaponeurosis [7]. In hernia surgery, the inguinal ligament is usually introduced as an attachment point of the conjoined tendon and transversus abdominis muscle-tendon to enhance the front and back wall of the inguinal canal [8]. Thus, the inguinal ligament has a relative fixed position and powerful tension. In addition, the portion of the inguinal ligament between the entrance of the inguinal canal and anterior superior iliac spine, especially for 1–2 cm distance from anterior superior iliac spine, presents two major characteristics: (1) a broad width with strong tension; (2) no large vessels and nerves around it. Thus, we hypothesized that the portion of the inguinal ligament, especially for 1–2 cm distance from anterior superior iliac spine, can be used as an anchor point for suspending the vaginal cuff.

Our previous study had confirmed that laparoscopic inguinal ligament suspension (LILS) could achieve excellent outcomes with higher anatomical and subjective cure rates and lower intra- and postoperative complications for vaginal vault prolapse [9]. As a new surgical technique for POP, a prospective randomized controlled trial comparing the LILS with standard technique of LSC is necessary. The aim of this study is to describe the rationale and design of a prospective randomized trial for comparing the efficacy, safety, and feasibility of LILS with LSC in the treatment of POP.

## Methods/Design

### Study objective

The aim of this randomized controlled trial is to compare LILS and LSC in the treatment of stage 2 or higher POP in terms of perioperative parameters, such as surgical time, blood loss, intraoperative complications, and hospital stay as well as objective and subjective success rates, recurrence of prolapse, functional recovery, quality of life, postoperative complications, sexual functioning, and cost-effectiveness.

### Hypothesis

Our hypothesis for this study is that there is no difference in anatomical success rate and patients’ satisfaction of the anterior, posterior, and apical compartment between laparoscopic inguinal ligament suspension and laparoscopic sacrocolpopexy in symptomatic women with uterine descent pelvic organ prolapse questionnaire (POP-Q) stage 2 or higher. Possibly, LILS may be associated with shorter surgical time, less blood loss, fewer intra- and postoperative complications, and more improvement of sexual functioning than LSC.

### Study design

**Fig 1:**
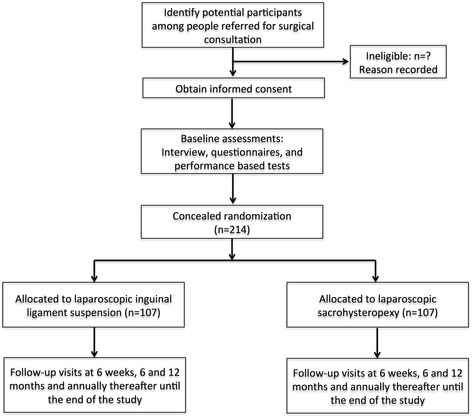
Study flow diagram

**Fig 2:**
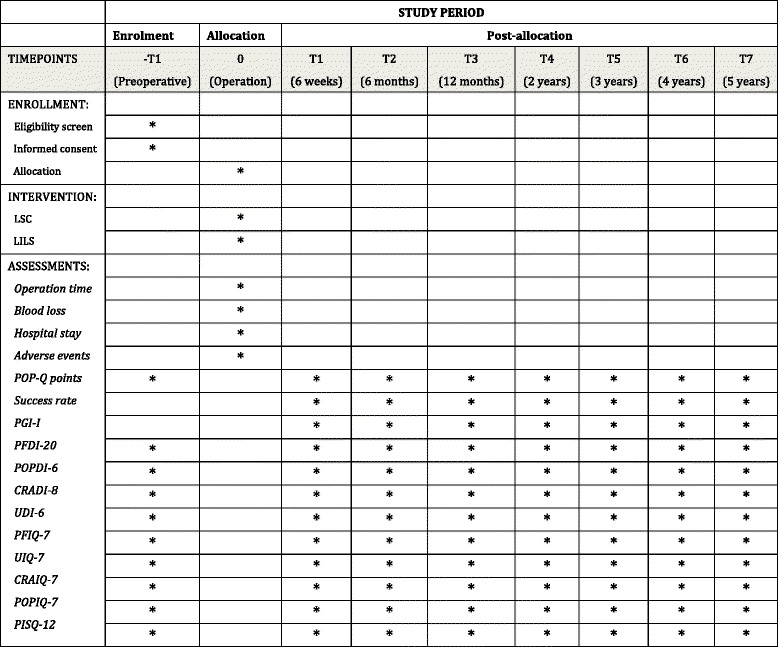
SPIRIT figure

The trial is a multicenter, prospective, randomized blinded clinical trial conducted with the aim to determine the noninferiority of the primary endpoint between LSC with LSC for POP. The trial has been registered on the China Trial Register (CTR) under the identifier ChiCTR-INR-15007408. Postoperative follow-up will be conducted after 6 weeks, 6 months, 12 months, and annually thereafter until 5 years. Patients will undergo a standard gynecological examination (including a POP-Q examination) and fill in questionnaires. A figure showing the planned visit and examination schedule was presented in Fig. 1. This study protocol was developed in accordance with the Standardized Protocol Interventions: Recommendations for Interventional Trials (SPIRIT) 2013 Statement (Additional file 1 for the completed SPIRIT checklist). The time schedule of enrollment, assessment, interventions, and follow-up are detailed in SPIRIT figure (Fig. 2).

### Study population and recruitment

All women seeking treatment for symptomatic POP with uterine descent POP-Q stage 2 or higher will be considered for inclusion in the trial. Patients with co-existing anterior/posterior defects or concomitant incontinence surgery can also be included.

Women with a surgical history for POP, known malignancy or abnormal cervical smears, a desire to preserve fertility, abnormal ultrasound findings of the uterus or ovaries or abnormal uterine bleeding, and contraindication for laparoscopic surgery are excluded from the study. Individuals who are unable to provide informed consent or to complete the testing or data collection are also excluded.

Eligibility is assessed by a gynecologist and/or residents of our hospital. Patients eligible for participation are counselled on the long duration of follow-up that is involved in the study. The risks associated with uterus removal are clearly outlined. Subsequently, women are provided with verbal and written information prior to enrollment, and written informed consent is obtained.

### Primary and secondary outcomes

The primary outcomes of this study included perioperative parameters such as surgical time, blood loss, intraoperative complications, hospital stay, and anatomical success rate using the POP-Q classification at 6 weeks, 6 months, 12 months, and annually till 5 years after surgery. The POP-Q system has been developed by the International Continence Society and is a reliable and reproducible method for assessing organ descent [10]. The lowest level of organ descent during Valsalva is measured relative to its distance from the hymen: points above the hymen are negative and those below are positive; vaginal support at the level of hymen is represented as ‘0 cm’. The anatomical success rate is defined as less than stage 1 (all vaginal sites at least 1 cm above the hymen on Valsalva), as scored by the POP-Q system. Secondary outcomes of this study include subjective outcome and improvement in general and disease-specific quality of life, short- and long-term complications, postoperative recovery, sexual functioning, and cost-effectiveness after LILS or LSC at each follow-up point.

### Randomization

After signing an informed consent form, patients are randomized in a 1:1 ratio to either LILS or LSC centrally using a computerized random number generated at the moment of inclusion by a non-physician blinded to the women’s history and with no contact with the patients. Randomization will be stratified according to severity of prolapse (POP-Q stage 2, 3, or 4). The details of the series will be withheld from the investigators or to the participating gynecologists and all participants will receive unique study numbers. The subjects will be informed about the allocated operative procedure shortly after the randomization. Those who withdraw after randomization will be treated according to the preference of the gynecologist.

### Data collection

All patients will undergo routine gynecological examination, which is part of the standard procedure before surgery. This examination includes pelvic ultrasound to exclude uterine or ovarian disease, routine cervical screening, and vaginal inspection in a 45° semi-upright position for staging uterovaginal prolapse by POP-Q examination on maximum Valsalva effort in the lithotomy position. Urodynamic testing will also be performed in patients with urinary incontinence.

Demographics, medical, and obstetric history, previous pelvic floor and gynecological surgery were collected using standardized questionnaires. The anatomical success rate as scored by the POP-Q system and subjective satisfaction rate according to the Patient Global Impression of Improvement Scale (PGI-I) scale that integrates bladder, bowel, prolapse, sexual function domain, severity, bothersomeness, and condition-specific quality of life were recorded [11]. Other questionnaires included symptom severity, quality of life, and sexual activity in accordance with the Pelvic Floor Distress Inventory (PFDI-20), Pelvic Floor Impact Questionnaire (PFIQ-7) and the Pelvic Organ Prolapse/Urinary Incontinence Sexual Questionnaire (PISQ-12) scores at baseline and postoperation at 12 months. The PFDI-20 and PFIQ-7 questionnaires consist of three subscales each. PFDI-20 reflects the different perspectives of bulging symptoms (Pelvic Organ Prolapse Distress Inventory-6, POPDI-6), bowel problems (Colorectal-Anal Distress Inventory-8, CRADI-8), and urinary symptoms (UDI-6: Urinary Distress Inventory-6, UDI-6). PFIQ-7 describes the different impacts on a woman’s social life related to urinary tract (Urinary Impact Questionnaire-7, UIQ-7), bowel or rectal (Colorectal-Anal Impact Qestionnaire-7, CRAIQ-7), and vaginal or pelvic symptoms (Pelvic Organ Prolapse Impact Questionnaire-7, POPIQ-7). PISQ-12 describes sexual function in relation to prolapse or urinary incontinence. High PFDI-20 and PFIQ-7 indicate impaired function, and a high PISQ-12 score indicates good sexual function [12].

Intraoperatively, patients are assessed for operative time, blood loss, and complications. The impact of bleeding is also assessed by the measurement of hemoglobin levels 24 h after surgery. During hospitalization and in the first 6 weeks after surgery, the patients are asked to keep a diary, which contains the following items: postoperative pain measured by the Visual Analogue Score (VAS), pain medication intake and the Recovery Index-10 (RI-10) recovery questionnaire. The RI-10 recovery questionnaire is a validated quality-of-life questionnaire measuring subjective postoperative recovery.

Postoperatively, patients will visit the hospital at 6 weeks (routine postoperative consultation), 6 months, 12 months, and yearly thereafter. The total duration of the follow-up period is 5 years. Each check-up point includes answering a standardized written questionnaire regarding symptom severity, quality of life, and sexual activity (similar to the questionnaires at baseline) and a clinical examination (including POP-Q). The long-term complications in both groups are recorded.

### Interventions

Eligible women will be randomly allocated to receive either LILS or LSC. To eliminate the learning curve effect, the operation will always be performed by the same term of surgeons with more experience of performing both procedures. The detailed surgical procedure is presented in Additional file 2.

### Laparoscopic inguinal ligament suspension (LILS)

Laparoscopic hysterectomy with/without bilateral salpingo-oophorectomy was performed. A meticulous double-layered cuff closure was performed at the center of the vaginal cuff with a number 1 Vicry suture. The anterior vaginal wall was dissected from the bladder down to the bladder neck and the dissection was repeated for the posterior vaginal wall, which was prepared down to the levator ani plane. Afterward, the short arm of a self-styled cross-shaped mesh (Aspide Medical, La Talaudière, France) was placed into the vesico-vaginal space and recto-vaginal space and sutured to the anterior and posterior vaginal wall with four polyglycolic 1-0 sutures, respectively. Then, a portion of the inguinal ligament located 1–2 cm distance from the anterior superior iliac area was exposed. An extraperitoneal tunnel along the round ligament between the suspension points and the vaginal vault was created. The long arms of the mesh were introduced outside the peritoneum along the round ligament to the inguinal ligament suspension points and then fixed into the inguinal ligament/fascia. Finally, the peritoneal incision was closed so as to place the suspension mesh outside the peritoneum.

### Laparoscopic sacrocolpopexy (LSC)

The initial procedure of LSC was performed similarly to the procedures on the vaginal wall, and sutures were applied on the anterior or posterior vaginal wall similar to LILS. In LSC, a rectangular macroporous, monofilament polypropylene mesh was attached to the anterior vaginal wall with four polyglycolic 1–0 sutures. Another rectangular polypropylene mesh was attached to the posterior vaginal wall with four polyglycolic 1–0 sutures. Then, the peritoneum over the sacral promontory will be incised; the right ureter will be identified. The mesh will be tacked from the vaginal vault to the sacral promontory by using 5.3 × 3.7 mm staples to elevate the vaginal stump. The peritoneum will be closed in a manner covering the promontory part of the mesh and with a running suture covering the cervical part of the mesh.

During the same procedure, additional anterior and/or posterior colporrhaphy or incontinence surgery can be performed if necessary, according to the standard procedures of the hospital.

## Statistical analysis

### Sample size and power considerations

The sample size for this trial was estimated based on the hypothesis that both interventions are equivalent in anatomical success rate within a 1-year follow-up. A review [13] including 12 publications [14–24] showed an objective success rate of 78% to 100% for LSC. Thus, the sample size was calculated based on objective success rates of 90% within 1 year. If a two-sided alpha level of 5% was applied, then 89 individuals per group would be required to achieve a power of 1-β = 80% for a noninferiority margin of 15%. The rate of loss to follow-up without recurrence of prolapse is expected to sum up to 20%. Finally, a total of 214 participants (107 for each group) will be needed. Anatomical success rate at each follow-up point assessed by a POP-Q-examination in both study groups will be considered as primary outcome. Surgical anatomical success rate is defined as less than stage 1 (all vaginal sites at least 1 cm above the hymen on Valsalva), as scored by the POP-Q system. Noninferiority of LILS to LSC will be concluded if the lower limit of the 95% confidence interval in anatomical success rates lies above the noninferiority margin of -15% [25].

### Data analysis

The analysis will be performed with the intention to treat, and stratified for severity of prolapse. Patient characteristics will be summarized using descriptive statistics for continuous variables (mean ± standard deviation, minimum, maximum, and sample size) and frequency tables for categorical variables (numbers and percentages). Differences in group means were tested by Student’s *t* test, or Wilcoxon rank-sum test where data failed to follow a normal distribution after testing with the Kolmogorov-Smirnov test for normality. Differences in group proportions were tested using a chi-square test, or a Fisher’s exact test if data were sparse. Subgroup analysis based on body mass index (BMI) (< 24, ≥ 24), presence of concomitant surgical procedures, or prolapse stage (stage 2, stage 3, and stage 4) will be performed to identify the potential indication for each technique. Statistical analysis was performed using SPSS 20 for Windows (IBM Corp., Armonk, NY, USA) with *p* < 0.05 considered as statistically significant.

### Ethics

The trial has been approved by the Medical Ethics Committee of Shanghai First Infant and Maternity Hospital and the local ethics committees of the participating centers (KS1513). Prior to randomization, informed consent will be obtained. A Data and Safety Monitoring Board (DSMB) consisting of two surgeons and one methodologist was estimated. The DSMB will perform regular reviews of all study outcomes and adverse event data after every 25 included patients to ensure that there is no difference in rates of complication and recurrence rates in either group. The result of the meeting will be relayed to the Trial Steering Committee. The advice of the DSMB will only be communicated to the sponsor of the trial. The results of the trial will be widely disseminated to patients, health professionals, commissioners, and policy makers. To achieve a wide clinical audience, the trial results will be disseminated at international congresses, and in peer-reviewed scientific journals.

## Discussion

This is a protocol for a randomized trial comparing LILS with LSC for the treatment of POP-Q stage 2 or higher prolapse with regard to perioperative parameters, such as surgical time, blood loss, intraoperative complications, and hospital stay as well as objective and subjective success rate, recurrence of prolapse, functional recovery, quality of life, postoperative complications, sexual functioning, and cost-effectiveness.

LSC has been viewed as the gold standard treatment for women with POP who desire reconstructive surgery [3]. Robust data are available as regards the practice of this procedure and hence provided cumulative information on the technique for treating POP [13]. The exposure of the anterior longitudinal ligament is the first surgical step of LSC. However, for patients with obesity, abdominopelvic adhesions, sigmoid megacolon, or anatomic variation of abdominopelvic organs, the procedure is relatively hazardous and time-consuming. It is in this anatomical area where most of bleeding and ureter injuries occur. LILS allowed skipping the dangers and technical challengers of sacral mesh fixation. Thus, we considered LILS to be suitable for patients who present with difficult access to the promontory [9]. In our previous study, we reported that LILS is an effective and safe method for treating vaginal vault prolapse.

The findings of this trial will contribute to answer the question whether LILS technique is preferable in women with symptomatic uterine prolapse POP-Q stage 2 or higher. If noninferiority in surgical success is found, the comparison of the secondary outcomes will be essential in selecting the preferred strategy.

## Trial status

This trial is ongoing. The study began in March 2016, and the primary follow-up is expected to be completed by March 2018.

## Additional files

Additional file 1: SPIRIT checklist: recommended items to address in a clinical trial protocol and related documents. (DOC 111 kb)

Additional file 2: The detailed surgical procedure for both techniques. (DOCX 96 kb)

## Abbreviations

CRADI-8: Colorectal-Anal Distress Inventory-8
CRAIQ-7: Colorectal-Anal Impact Questionnaire-7
DSMB: Data and Safety Monitoring Board
LILS: Laparoscopic inguinal ligament suspension
LSC: Laparoscopic sacrocolpopexy
PFDI-20: Pelvic Floor Distress Inventory-20
PFIQ-7: Pelvic Floor Impact Questionnaire-7
PGI-I: Patient Global Impression of Improvement Scale
PISQ-12: Pelvic Organ Prolapse/Urinary Incontinence Sexual Questionnaire-12
POP: Pelvic organ prolapse
POPDI-6: Pelvic Organ Prolapse Distress Inventory-6
POPIQ-7: Pelvic Organ Prolapse Impact Questionnaire-7
RI-10: Recovery Index-10
UDI-6: Urinary Distress Inventory-6
UIQ-7: Urinary Impact Questionnaire-7
