# Supplementary material for: Laparoscopic inguinal ligament suspension versus laparoscopic sacrocolpopexy in the treatment of pelvic organ prolapse: study protocol for a randomized controlled trial
Source: Trials. 2018 Mar 5;19:160. doi: 10.1186/s13063-018-2494-x (PMC5838885; doi:10.1186/s13063-018-2494-x)
Supplement: Supplementary file 2 — The detailed surgical procedure for both techniques. (DOCX 96 kb) [file 13063_2018_2494_MOESM2_ESM.docx]

**Interventions**

Eligible women will be randomly allocated to receive either a l rial to compare the efficacy, aaparoscopic inguinal ligament suspension or laparoscopic sacrocolpopexy. Hysterectomy was performed if necessary. All procedures will be performed under general anesthesia or spinal analgesia according to the preference of patient and anesthesiologist. All women receive perioperative antibiotics and thrombosis prophylaxis. Postoperatively a bladder catheter is placed and removed according to local hospital protocol. Patients will receive analgesics if necessary in accordance with local hospital protocol. All patients are advised to abstain from heavy physical work for a minimal period of 6 weeks.

*L rial to compare the efficacy, aaparoscopic inguinal ligament suspension*

The procedure was similar with our previous study. In brief, a pneumoperitoneum was created, and four laparoscopic ports were placed: one 10-mm umbilical port, one 10-mm lateral port at the umbilical port and two 5-mm lateral ports at the suprapubic level. Then, a total laparoscopic hysterectomy with/without bilateral salpingooophorectomy was performed. A meticulous double-layered cuff closure in the center of the vaginal cuff with number 1 Vicry was done. The anterior vaginal wall was dissected from the bladder down to the bladder neck to expose a large vaginal wall area and the dissection was repeated for the posterior vaginal wall, which was prepared down to the levator ani plane. Following that, the short-arm of a self-styled “**+**” shaped mesh (Aspide Medical, France) was placed into vesico-vaginal space and recto-vaginal space and sutured to anterior or posterior vaginal wall, while a vaginal examination was carried out at the same time to ensure that no mesh exposure occurred. Then, the round ligament of the uterus was used as anatomic landmark for an area of inguinal canal and anterior superior iliac spine was identified as a landmark for original of inguinal ligament. The suspension area of inguinal ligament between the inlet of inguinal canal and anterior superior iliac spine was identified. The portion of inguinal ligament that was 1-2 cm distance from anterior superior iliac was exposed completely. An extraperitoneal tunnel along the round ligament between the suspension points and the vaginal vault was created. The long-arms of mesh were introduced outside peritoneum along the round ligament to the inguinal ligament suspension points and fixed into the inguinal ligament/fascia. At last, the peritoneal incision was closed so as to place the suspension mesh outside the peritoneum.

*Laparoscopic* *sacrocolpopexy*

Four laparoscopic ports (umbilical, suprapubic, two lateral ports) will be placed and a pneumoperitoneum will be created. Then, a total laparoscopic hysterectomy with/without bilateral salpingooophorectomy was performed. A meticulous double-layered cuff closure in the center of the vaginal cuff with number 1 Vicry was done. The anterior vaginal wall was dissected from the bladder down to the bladder neck to expose a large vaginal wall area and the dissection was repeated for the posterior vaginal wall, which was prepared down to the levator ani plane. A rectangular macroporous, monofilament polypropylene mesh was attached with four polyglycolic 1–0 sutures and another rectangular polypropylene mesh was attached to the posterior vaginal wall with four polyglycolic 1–0 sutures, avoiding the levator ani. Then, the peritoneum over the sacral promontory will be incised; the right ureter will be identified. The mesh will be tacked fro the vaginal vault to the sacral promontory using 5.3 × 3.7 mm staples to elevate the vaginal stump. The peritoneum will be closed covering the promontory part of the mesh and a running suture covering the cervical part of the mesh.

mfo the vaginal vaultinal wall, During the same procedure, additional anterior and/or posterior colporrhaphy or incontinence surgery can be performed if necessary, according to the standard procedures of the hospital.
